# Characteristics determining host suitability for a generalist parasite

**DOI:** 10.1038/s41598-018-24627-1

**Published:** 2018-04-19

**Authors:** Bård G. Stokke, Irja I. Ratikainen, Arne Moksnes, Eivin Røskaft, Karl Schulze-Hagen, David I. Leech, Anders Pape Møller, Frode Fossøy

**Affiliations:** 1 Department of Biology, Faculty of Natural Sciences and Technology, Norwegian University of Science and Technology (NTNU), Høgskoleringen 5, NO-7491 Trondheim, Norway; 2 Norwegian Institute for Nature Research (NINA), P.O. Box 5685 Sluppen, NO-7485 Trondheim, Norway; 3 Bleichgrabenstr, 37, D-41063 Mönchengladbach, Germany; 4 British Trust for Ornithology, The Nunnery, Thetford, IP24 2PU UK; 5 Ecologie Systématique Evolution, Université Paris-Sud, CNRS, AgroParisTech, Université Paris-Saclay, F-91405 Orsay, Cedex France

## Abstract

Host quality is critical for parasites. The common cuckoo *Cuculus canorus* is a generalist avian brood parasite, but individual females show strong preference for a specific host species. Here, we use three extensive datasets to investigate different host characteristics determining cuckoo host selection at the species level: (i) 1871 population-specific parasitism rates collected across Europe; (ii) 14 K cases of parasitism in the United Kingdom; and (iii) 16 K cases of parasitism in Germany, with data collected during the period 1735–2013. We find highly consistent effects of the different host species traits across our three datasets: the cuckoo prefers passerine host species of intermediate size that breed in grass- or shrubland and that feed their nestlings with insects, and avoids species that nest in cavities. Based on these results, we construct a novel host suitability index for all passerine species breeding in Europe, and show that host species known to have a corresponding cuckoo host race (gens) rank among the most suitable hosts in Europe. The distribution of our suitability index shows that host species cannot be classified as suitable or not but rather range within a continuum of suitability.

## Introduction

Parasites are strongly dependent on host suitability for successful reproduction. Some parasites may prosper using a broad range of hosts, while others specialise on one particular host species^1^. For generalist parasites, it is very important to select host species that maximize their fitness. Studies on host species preferences show variable degrees of preference in different systems (e.g.^2–4^), and significant proportions of parasite host range is currently unknown in many systems^5^. Disentangling the characteristics determining host use in parasites is important for understanding host-parasite coevolution and the evolution of host-specific adaptations in their ongoing arms races, but also for understanding emerging diseases and invasion success of pathogens exploiting crops, domestic animals and human beings. Knowledge of the importance of various host life history traits for successful parasite utilization would for instance allow predictions concerning the host potential for species in geographical areas into which parasites may expand, either in relation to climate change scenarios or in a biological invasion framework^6,7^.

Avian brood parasites exploit the parental care of their avian hosts, often at the expense of the hosts’ own reproductive output. The common cuckoo *Cuculus canorus* is a generalist avian brood parasite that is widely distributed throughout Eurasia and is known to have utilized at least 125 passerine species in Europe alone^8–10^. Individual female cuckoos, however, are considered host-specific and preferentially lay in nests of one, or a few, host species, which then incubate their eggs and raise the parasitic chick^11–18^. Individual cuckoos can therefore be classified into host races, termed gentes, of which several can be recognized based on their egg phenotype, and which often mimics the egg phenotype of their host^19–21^, but not always^10^. More than 200 passerine species breed in Europe^22^ but interestingly, fewer than 20 cuckoo gentes have so far been described within this area^8–10,23,24^, implying that many potential hosts are not regularly used by cuckoos^10,25–29^. Female cuckoos belonging to a specific gens may sometimes lay eggs in the nests of hosts other than the target species^11,30^, and probing of new hosts and host switching is likely to be an important mechanism for the evolution of new gentes^12,25,31–34^. The use of a particular host species may therefore change through time; several passerine species show strong anti-parasite adaptations without being parasitized at present, indicating that they have been used as cuckoo hosts in the past^35–40^.

Data on cuckoo parasitism clearly show that host use varies among habitats^26,41,42^. In addition, parasitism within a particular host population is not random, but depends on specific variables related to nest structure and placement, phenology and host behaviour, quality and density^24,43–55^. The relative importance and generality of each specific characteristic that makes a potential host species prone to parasitism is, however, not well known. Such information is pivotal for understanding the potential host range of parasite species.

Many factors have been suggested to affect the suitability of passerine species as cuckoo hosts: diet, nest placement, habitat structure, abundance, duration of egg and nestling period, body size (as a proxy of egg size), nest size and timing of their breeding season^9,27,34,35,41,56–63^. Glue and Murray^64^ added “tolerance of the host to disturbance and egg mimicry” as another important attribute of a suitable host.

However, few studies have analysed the factors explaining variation in host use by the cuckoo by using individual-level data across host species. Soler *et al*.^65^ found that cuckoo parasitism in UK was more prominent in potential host species with a relatively short nestling period, host species building open nests, and host species with large populations. However, host species with large populations were not more prone to parasitism in southern Finland^13^. Soler *et al*.^66^ found that cuckoo gentes with highly mimetic eggs were most often found in hosts with large population sizes and with low spatial variation in abundance across countries, suggesting that large host populations are important for stable long-term co-evolutionary interactions.

Here, we present three unique datasets with one dataset on 1871 population-specific parasitism rates across European passerines and two datasets on 14 K and 16 K host species-specific records of parasitism in UK and Germany, respectively. These two geographical entities are ideal for analysis of host use because of long-term collections of data on parasitism available from museum collections, literature and various ringing and nest record schemes. This approach allows us to evaluate the generality of the results by determining whether the same factors are identified as important predictors of parasitism in the three independent datasets. We investigate seven specific host life-history traits (nestling food, adult body size, nest placement, habitat, overlap in breeding period with the cuckoo, nest cup depth, and nest height above ground) that may all influence suitability as cuckoo hosts among passerine birds as suggested by previous studies. Finally, we use the population-specific parasitism rates to calculate a host suitability index and predict the suitability of all passerine bird species breeding in Europe.

## Results

**Figure 1:**
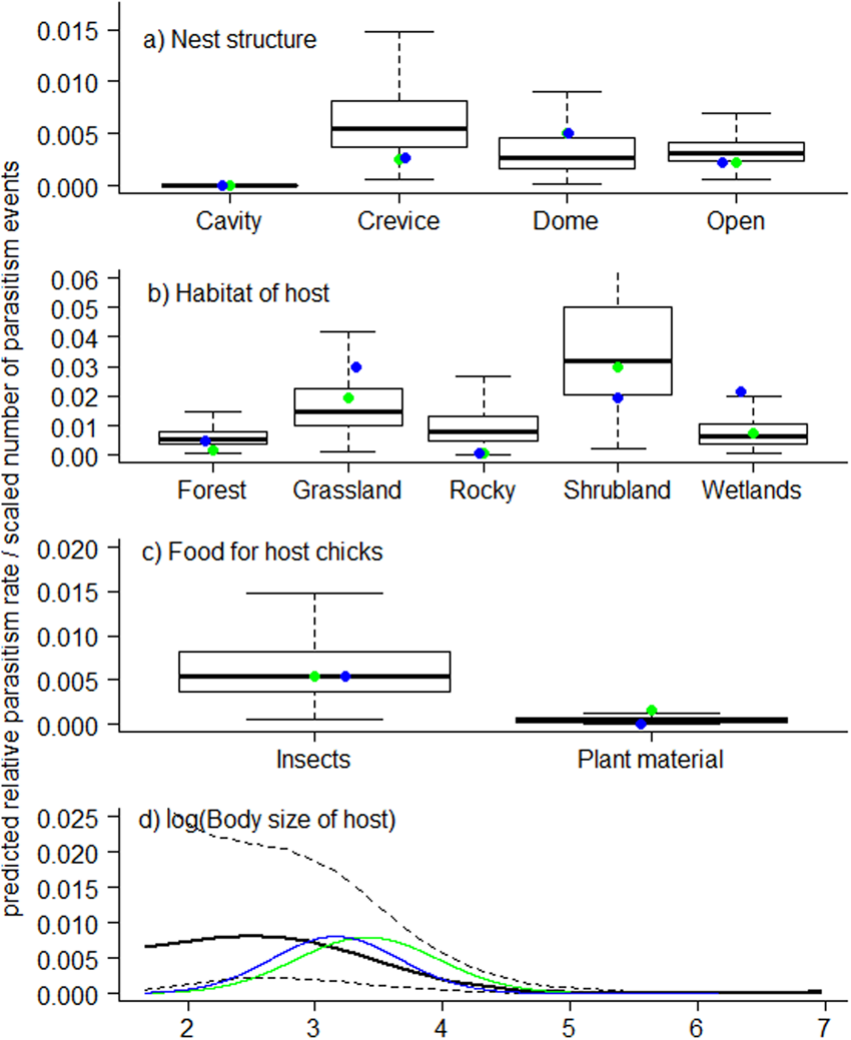
Predicted effects of ecological variables on population-specific parasitism rates (black) and number of parasitism events by cuckoos in UK (green) and Germany (blue) based on (**a**) nest structure (**b**) habitat of host (**c**) food type the host provide for its young and (**d**) the body size of the host. Note that values for number of parasitism cases in UK and Germany are scaled for easier comparison with parasitism rates and are not originally on the scale of the y-axis. Box blots show the mean predicted relative parasitism rate, outer box show the first and third quantile and the dotted lines show the 95% credible limits. See Materials and Methods for further explanation on how these predicted values were obtained.

Models based on the two independent datasets describing the number of parasitism cases in Germany and the UK and the dataset with parasitism rates across Europe show remarkably similar results: (i) species that nest in cavities are used less frequently than those with other nest locations (Fig. 1a and Table 1); (ii) species breeding in forest and rocky areas are used less than species breeding in other habitats (Fig. 1b, Tables 1 and 2); (iii) species feeding their nestlings with plant material are used less frequently than species feeding their nestlings with insects, although not significantly so in the UK (Fig. 1c, Tables 1 and 2); (iv) species with either larger or smaller body size are used less than species with intermediate sizes (Fig. 1d, Tables 1 and 2); and (v) species with smaller population sizes have fewer parasitism events than species with larger populations both in the UK and Germany (Table 1). However, from the current analyses we are not able to tell if there is a deviation from what would be expected if hosts are being used at random as expected from population size. Nest height, nest depth and overlap in breeding period do not affect parasitism in any of the three datasets (Tables 1 and 2). We also note that results are qualitatively and quantitatively very similar if we include all populations regardless of number of nests or if we exclude all populations with less than ten nests from our analysis.

**Table 1:** Factors explaining variation in cuckoo parasitism rates among passerine species in Europe.

	Estimate	SE	p
Intercept	−16.416	4.428	0.000
log(nest.depth + 1)	−0.206	0.954	0.829
Crevice vs. Cavity	**7.047**	**1.230**	**0.000**
Dome vs. Cavity	**6.405**	**1.329**	**0.000**
Open vs. Cavity	**6.517**	**1.174**	**0.000**
log(Nest height + 1)	0.001	0.095	0.988
Grassland vs. Forest	1.003	0.604	0.097
Rocky areas vs. Forest	0.380	0.980	0.698
Shrubland vs. Forest	**1.757**	**0.462**	**0.000**
Wetlands vs. Forest	0.158	0.660	0.811
Plant material vs. Invertebrates	−**2.694**	**0.678**	**0.000**
log(Female body size)	3.556	2.360	0.132
log(Female body size^2^)	**−0.689**	**0.347**	**0.047**
Breeding overlap	0.001	0.013	0.901

Parameter estimates are from a binomial regression model of population-specific parasitism rates with species identity included as random factor. Estimates in bold have p-values below 0.05 and are considered significant. See Materials and Methods for details regarding each factor.

**Table 2:** Ecological factors explaining variation in cuckoo parasitism among passerine species in UK and Germany.

Count model coefficients	UK			Germany		
	Estimate	SE	p	Estimate	SE	p
Intercept	−20.030	4.742	0.000	−27.758	6.054	0.000
log (nest.depth + 1)	−0.330	0.885	0.709	−0.995	1.707	0.560
Crevice vs. Cavity	**4.707**	**0.730**	**0.000**	**3.750**	**1.147**	**0.001**
Dome vs. Cavity	**5.405**	**1.140**	**0.000**	**5.033**	**1.228**	**0.000**
Open vs. Cavity	**4.505**	**0.777**	**0.000**	**3.766**	**1.070**	**0.000**
log(Population size + 1)	**0.283**	**0.067**	**0.000**	**0.524**	**0.090**	**0.000**
log(Nest height + 1)	−0.041	0.131	0.756	0.257	0.168	0.125
Grassland vs. Forest	1.105	0.575	0.055	**1.770**	**0.868**	**0.041**
Rocky areas vs. Forest	−1.228	1.272	0.334	−1.764	1.172	0.132
Shrubland vs. Forest	**1.596**	**0.508**	**0.002**	**1.933**	**0.816**	**0.018**
Wetlands vs. Forest	**3.074**	**0.752**	**0.000**	**3.177**	**1.163**	**0.006**
Plant material vs. Invertebrates	−1.194	0.624	0.056	−**4.326**	**0.907**	**0.000**
Breeding overlap	−0.008	0.022	0.721	0.023	0.025	0.345
log(Female body size)	**10.625**	**2.487**	**0.000**	**13.683**	**3.207**	**0.000**
log(Female body size^2^)	−**1.544**	**0.367**	**0.000**	−**2.146**	**0.465**	**0.000**
Log(theta)	−0.291	0.224	0.194	−0.971	0.283	0.001
**Zero hurdle model coefficients**						
Intercept	−5.696	14.438	0.693	−20.092	7.837	0.010
log(nest.depth + 1)	−1.939	2.110	0.358	1.144	1.095	0.296
Crevice vs. Cavity	4.252	2.353	0.071	**3.650**	**1.610**	**0.023**
Dome vs. Cavity	0.069	2.703	0.980	**3.972**	**1.870**	**0.034**
Open vs. Cavity	5.489	3.157	0.082	**4.148**	**1.562**	**0.008**
log(Population size + 1)	**0.895**	**0.385**	**0.020**	**0.543**	**0.143**	**0.000**
log(Nest height + 1)	−1.359	1.313	0.301	0.027	0.209	0.896
Grassland vs. Forest	9.059	5.601	0.106	3.307	2.933	0.260
Rocky areas vs. Forest	−0.144	2.264	0.949	−1.540	1.450	0.288
Shrubland vs. Forest	12.633	6.433	0.050	0.391	0.870	0.653
Wetlands vs. Forest	−4.319	2.932	0.141	−0.199	1.316	0.880
Plant material vs. Invertebrates	16.245	4479.8	0.997	−**2.481**	**1.146**	**0.030**
Breeding overlap	0.078	0.063	0.213	−0.029	0.029	0.319
log(Female body size)	3.798	5.730	0.508	**7.897**	**3.925**	**0.044**
log(Female body size^2^)	−0.919	0.853	0.281	−**1.249**	**0.573**	**0.029**

The estimates presented are from hurdle regression models with two components: a truncated negative binomial component for the positive counts (Count model coefficients) and a binomial component for the zero vs positive counts (Zero hurdle model coefficients). Estimates in bold have p-values below 0.05 and are considered significant. See Materials and Methods for details regarding each factor.

**Figure 2:**
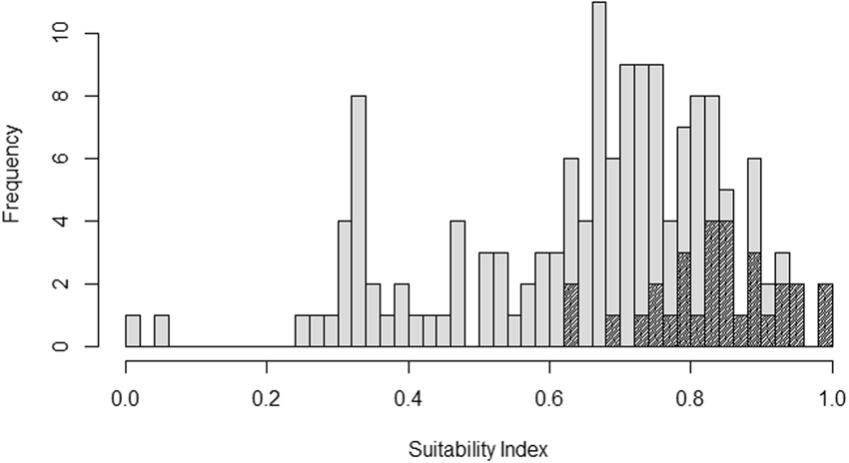
Frequency plot of the host suitability index for passerine birds in Europe. Hatched bars = species with a corresponding cuckoo host race (gens) light grey bars include all passerine species in Europe.

**Figure 3:**
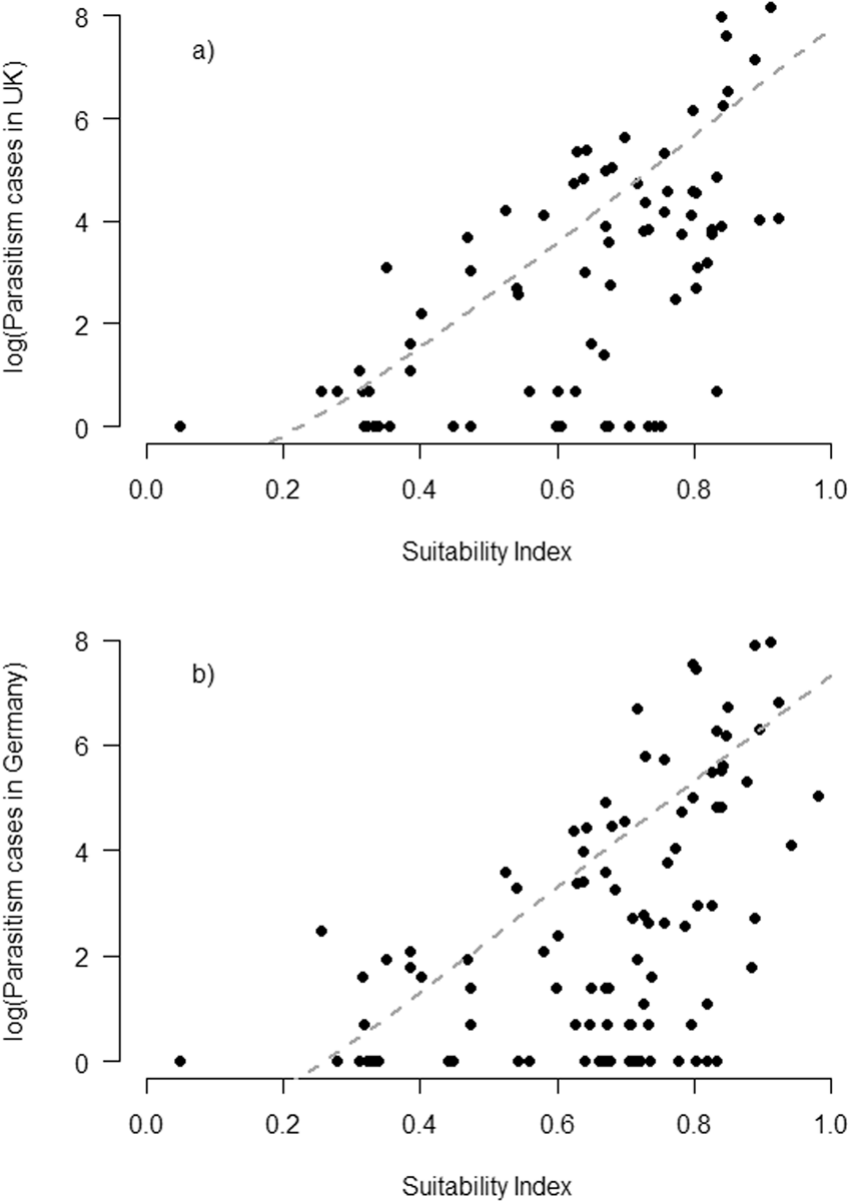
Fit between our host suitability index based on European population-specific parasitism rates and number of parasitism events recorded in (**a**) UK (R^2^ = 0.35) and (**b**) Germany (R^2^ = 0.28). The grey dotted lines are predicted number of records for increasing suitability along the x-axis. This line is based on predictions from hurdle models (see main text), including only the suitability index as explanatory variable and recorded parasitism events as response variable in each of the two countries. Only species that are recorded in the specific country are included.

The distribution of our host suitability index calculated for all European passerine species does not show a clear bimodal separation between suitable and unsuitable host species, but rather a continuum from low to high suitability (electronic supplementary material, Table S1 and Fig. 2). However, all species with a recognized corresponding cuckoo gens are ranked towards the high suitability end of the index (Fig. 2). Furthermore, the host suitability index, which is based on the model of parasitism rates across Europe, shows a strong correlation with the number of parasitism events in both Germany and the UK (Fig. 3a,b).

## Discussion

Species vary in their quality as hosts for parasites, as manifested through host-specific variation in parasite reproductive success^3,67^. Such variation in parasite success is also evident in avian brood-parasite systems^68^ and brood parasites should selectively target hosts that maximize the probability of successful fledging of the parasitic chick. Since there is pronounced variation in utilization among potential hosts, selection by cuckoos is clearly not random^25,26,28^. We find highly similar effects of the different ecological host traits on cuckoo parasitism in our three independent datasets. According to our results, the cuckoo prefers host species of intermediate size that feed their nestlings with insects, and tends to avoid species that nests in cavities or breed in forest or rocky areas, but we find little effect of nest height, nest depth and breeding overlap. Importantly, there is no single variable explaining host use by cuckoos, but rather a combination of variables that together influence parasitism rates.

Host body size is clearly important for host selection by cuckoos. Intermediate-sized passerines are in general parasitized at higher rates than smaller or larger species. Use of the smallest passerines may be hampered by inefficient incubation of the parasitic egg and inadequate provisioning of the parasitic chick. The largest passerines may be avoided for the same reasons, and in addition large host nests, eggs and chicks may render it difficult for the cuckoo chick to evict potential competitors. However, nest cup depth was not an important predictor of cuckoo parasitism in our analyses, despite a deeper or steeper-sided nest tending to render eviction more difficult^62,69^. Experiments have shown that cuckoo chicks growing up together with host chicks suffer significantly lower probability of survival than when raised alone^69–71^, but see^72^. It is possible that the combined effect of larger eggs and nest steep-sidedness would render the largest passerines unsuitable as cuckoo hosts.

The cuckoo chick is dependent on invertebrate food, and seed eating species have therefore been considered unsuitable as cuckoo hosts e.g.^10^. Nevertheless, species like greenfinch *Carduelis chloris* and linnet *C. cannabina* rank among the 10 most commonly used hosts in UK, and there was no significant effect of nestling food on parasitism in UK (Table 1). Greenfinches have been able to raise cuckoo chicks^73^, but this observation alone is not sufficient to conclude that they are high quality hosts, as we do not know the condition of the fledgling cuckoos and hence the likelihood of recruitment of cuckoos raised by greenfinches. Furthermore, none out of 20 cuckoo eggs in linnet nests recorded in the BTO Nest Record Scheme resulted in successfully fledged cuckoo chicks^25^. In contrast to UK, our data disclose that few cuckoo eggs have been found in this abundant species in Germany. Hence, the most plausible explanation for the relatively high use of seed eaters in UK is “mislaid” eggs by cuckoos belonging to other tribes. The dunnock *Prunella modularis*, one of the favourite hosts in UK but less so in Germany, often nests in similar habitats like linnets and greenfinches, and with similar nests and nest sites. Hence, we agree with Davies^10^ that dunnock cuckoos are most likely responsible for many of the parasitic eggs ending up in finch nests.

Species nesting in cavities often have small entrance holes and deep nests^74^. The small entrance hole poses great problems for the female cuckoo attempting to successfully place her egg into the nest cup^74^ and, even if she succeeds, her chick may grow too big to escape and become trapped inside. Chicks may also struggle to evict competing eggs and nestlings. Cavity nesters are therefore regarded as unsuitable hosts^35,71,75^, a prediction confirmed by our results.

Habitat showed a strong relationship with the number of parasitism events in all three datasets. Species breeding in shrubland and grassland were preferred by the cuckoo whereas species breeding in forests and rocky habitats were largely avoided. Wetland species were utilized relatively frequently in Germany, but not in the two other datasets. The cuckoo is dependent on high vantage points from where it can search for available host nests^58^, which may render species breeding in rocky areas unsuitable in most cases. Moreover, most of the species in UK and Germany breeding in rocky areas have small population sizes/densities, potentially making it more difficult for cuckoos to maintain a viable population. Many potential host species breeding in forest habitat are cavity breeders (like tits, Paridae) and of larger size (like thrushes, Turdidae), which may explain the relatively less use of forest breeding species than those breeding in other habitats. Wetland breeding species are apparently more used in Germany than in UK, which seems to be due to utilization of great reed warblers *Acrocephalus arundinaceus* in Germany, a species that is absent from UK.

Cuckoos are dependent on the ability to synchronise the timing of their breeding with that of their hosts. However, our analyses did not provide a significant effect of overlap in breeding season between the potential host species and the cuckoo. The reason for this could simply be that most passerine species overlap in duration of breeding season, and those with a small overlap in breeding season are generally large species or cavity breeders and therefore not suitable anyway.

Nest height above ground was also not a significant predictor of cuckoo parasitism in our analyses. This is contrary to the findings of Martín-Vivaldi *et al*.^76^, who suggested that cuckoos have difficulties finding host nests on the ground. They found lower egg rejection in ground-nesting passerines and hence concluded that ground-nesters are rarely used by cuckoos. Several cuckoo gentes, however, utilize hosts breeding on the ground. In the UK for instance, ground-nesting meadow pipits *Anthus pratensis* are among the most common hosts^41^. Moksnes and Røskaft^9^ mention *Anthus*, yellow wagtail, white wagtail, blue and *Emberiza* cuckoo egg morphs being found in ground-nesters. Previous within-species analyses are also in line with our findings; nest height was not a predictor of parasitism in marsh *Acrocephalus palustris* and reed warblers *A. scirpaceus*^24,77^.

There is considerable interspecific variation in the ability of hosts to recognize and reject cuckoo eggs e.g.^35,37^, which may influence our estimation of parasitism rates. In host species with well-developed egg rejection abilities, a poor mimic may be removed before its presence could be detected in the nest. This plausible scenario may lead to an underestimation of relative parasitism in such species compared to hosts that are poor rejecters. The data available in the present study, except those eggs stored at museums, do not allow us to assess egg mimicry since there is no description of egg appearance of either host or parasite in most sources. Variation in egg rejection among species may therefore blur the apparent suitability of different species over time. This could potentially make it harder for us to detect the factors important for host suitability, but is unlikely to contribute to false positive effects in our analyses. While some suitable hosts may not have been identified and hence misclassified in our suitability index, there is no reason to suspect an equivalent bias towards detection of parasitism in unsuitable hosts, because rejection behaviour is likely to have been selected for due to historic parasitism.

Our host suitability index based on population-specific parasitism rates correlated well with the number of parasitism events both in Germany and UK. In both countries, we also observed that few species were used more than would be expected by their suitability. On the other hand, quite a few species are being used less than predicted purely by the host suitability index. Although one should always be careful in the interpretation of variables based on estimates from statistical analyses, this bias suggests that the factors we have investigated may together act to modulate the suitability of species as cuckoo hosts. There may also be other limitations that we have not been able to detect with the current dataset, however, such as local variation in population sizes and rejection ability. Despite these possible caveats, the strong correlation between the host suitability index and the number of parasitism events suggest that it is useful as a species level index of host suitability among European passerines. When we then look at the distribution of this index among species (Fig. 2), it becomes clear that it would be too simplistic to regard species being either suitable or not as hosts for the cuckoo, but rather that the various species show various degrees of suitability. The host species with a corresponding cuckoo gens (classified based on egg mimicry) are all, as expected, placed among the most suitable hosts. According to the index there are a fair number of additional species that appear to be suitable for parasitism, but apparently without any gens attached to them. There are several possible explanations for this pattern. Firstly, some of these species only have very small population sizes in Europe, rendering them unsuitable as cuckoo hosts here but not necessarily in areas where they are more abundant. Little buntings *Emberiza pusilla* and Blyth’s reed warblers *Acrocephalus dumetorum*, for instance, are regularly parasitized in parts of Russia by cuckoos laying mimetic eggs^78,79^. Secondly, host use in some areas of Europe is poorly known, especially the southern and eastern parts. Hence, gentes that are still unknown to us may exist, such as cuckoos targeting those *Sylvia* warblers with a southern distribution (e.g.^80,81^). Thirdly, some cuckoo gentes, e.g. the dunnock gens, do not mimic the eggs of their hosts^10^. Hence, a classification based on egg appearance alone would result in missing some of the existing cuckoo gentes (e.g.^82^). Finally, as stated above, even though we have included many factors of importance for cuckoo host selection in constructing the host suitability index, there may still be others.

In many systems with generalist parasites, like ecto- or endo-parasites, the parasite is limited by dispersal between species. This is clearly not the case for the cuckoo and most other brood parasites. The bitterling *Rohdeus sericeus* is a parasitic fish that shares many of the same attributes as avian brood parasites and a similar pattern emerges for their host use. Investigations of four of their potential mussel hosts (*Anodonta anatine, A. cygnea, Unio pictorum* and *U. tumidus*) reveal differential suitability of the different host species, with the most suitable host offering twice as high survival for embryos as compared to the least suitable species of the four, and the two other species offer intermediate survival probabilities^3^. Furthermore, the bitterling prefers the four different hosts in the exact same order as their suitability^4,83^. The brown-headed cowbird *Molothrus ater* is, like the common cuckoo, a generalist brood parasite. In a study of nests of 34 potential host species, 18 were parasitized by cowbirds and a large range of parasitism frequencies were observed^84^. This suggests that even though a range of hosts can be used by generalist brood parasites, they are used in different frequencies according to factors that affect their suitability. In general, the nature of suitability indices will depend on parasite requirements. In the present analyses, we have selected host characteristics that have been hypothesized to explain variation in host use by the common cuckoo. For other parasites, there may be additional host traits that could be of importance in this sense (e.g. intraspecific variation in size and morphology and interspecific variation in coloniality^85^), and obviously the suitability index would also depend on the level of host specialization of the parasite.

In this study, relying on three novel large datasets, we have disclosed characteristics of potential hosts that may be important for cuckoo host selection at the species level. Host body size, nest structure, habitat and food type for the chicks are all important predictors of cuckoo parasitism, either independently or in combination. The same set of predictors explained variation in host use both in Germany and the UK, even though the actual species used varied somewhat between the two countries. Our findings offer a basis for more thorough analyses of temporal and spatial variation in cuckoo host use. We have shown that the relative importance of a suite of host characteristics on parasite utilization can be modelled statistically by using data from a subset of hosts in specific geographical areas. The outputs from such exercises can then be used to construct host suitability indices on a larger geographical scale for a larger set of species with unknown status as hosts, but where data on life history traits can be retrieved. Our results also demonstrate that potential cuckoo hosts should not distinctly be considered suitable or unsuitable, but rather be placed on a suitability continuum, with the majority of species located towards the more suitable end. We may predict similar patterns in other generalist parasites: many host traits going into their suitability index and similar distributions for the suitability of potential host species. More generally, such suitability indices may be valuable for predicting the potential for host use (current and future) in a whole range of host-parasite systems. This may be increasingly important for understanding species interactions in a world where both parasites and their potential hosts may have to shift their ranges due to climate change or human induced alterations of landscapes.

### Ethics

The data used in this study were entirely retrieved from the literature, museum collection, databases, etc.

## Material and Methods

Population-specific parasitism rates and cases of parasitism (cuckoo egg or chick) were obtained through various literature search, resulting in data ranging from the period 1735–2013 with the majority of cases from 1850 onwards, originating from more than 7,000 publications meticulously browsed by BGS (ISI Web of Science and Biodiversity Heritage Library, Google Scholar and the Natural History Museum library in Tring, UK, communication with British and German ornithologists, ringing and nest record schemes, museum egg collections and unpublished notes or reports stored in libraries and museums).

Firstly, we investigated 1871 population-specific parasitism rates from 139 passerine species, collected across Europe (10.5061/dryad.9r0n681). We only included parasitism rates based on a minimum of five nests (including parasitized and non-parasitized nests). Although we find five nests to be an appropriate cut-off for the number of nests needed to qualify as a population in these analyses, the number is not based on previous knowledge. We have therefore also undertaken the analyses with (1) all data included regardless of sample size, and (2) populations with ten or more nests included.

Secondly, we investigated 16,515 cases of parasitism from 100 passerine species in Germany and 14,507 cases of parasitism from 78 passerine species in UK (10.5061/dryad.9r0n681). One potential bias using these data is that parasitism rates are generally overestimated because populations that are likely to be parasitized are also more likely to be investigated for parasitism. However, our main question does not relate to actual parasitism rates, but rather to how host life-history traits affect relative parasitism rates, and we have no reason to believe that parasitism rates are overestimated relatively more for species with specific ecological characteristics.

The dataset on population-specific parasitism rates contains 2696 cases from UK and 2660 cases from Germany that are also included in the “cases of parasitism” datasets from UK and Germany. On the other hand, the dataset on population-specific parasitism rates includes additional cases from UK and Germany, where parasitism rates were reported to be zero (these are of course not included in the “cases of parasitism” datasets).

We selected the following variables as predictors of variation in parasitism between species:

1. Nest cup depth: Inner height of nest cup from bottom to rim (cm)^86^;
2. Nest structure: Classification of main nest structure. Four categories: (a) open, (b) crevice, (c) dome, or (d) cavity^87^.
3. Nest height above ground: Mean height of nest above ground or water (cm)^87^.
4. Habitat: Classification of main breeding habitat. Five categories: (a) wetlands, (b) shrubland, (c) forest, (d) grassland, or (e) rocky areas^22,87^.
5. Diet: Classification of main food source brought by host parents. Two categories; (a) animal or (b) plant material^87^.
6. Body size: Mean female body size (g)^87^.
7. Breeding overlap: Number of days that overlap with cuckoo breeding period. Breeding periods were extracted from^87^.
8. Population size: Number of breeding pairs in Germany and UK (mean estimate from BirdLife International^22^).

Using the dataset on population-specific parasitism rates in Europe, we ran a binomial generalised linear mixed effects model with counts of parasitized and unparasitised nests as response variable, using the glmer function in the lme4 package^88^ in R^89^. In this mixed model, we included species as a random effect. We have chosen not to include any phylogenetic effects in our analysis, because we assume that cuckoo host preferences in Europe were established after most passerine species evolved, and, therefore, do not expect cuckoo parasitism rates to be affected by the phylogeny of the species, but only by their actual trait values. Closely related species may have similar parasitism rates, but we believe that is due to similarities in their ecology rather in their phylogenetic history. We analysed predictor variables 1–7 listed above and additionally included the square of bird size to allow for a non-linear relationship, as we expected that species could potentially be both too small and too large to be suitable as hosts for the cuckoo. Generally, fixed factors were not markedly correlated, but nest height and habitat type grassland showed a correlation of 0.38, while body size and domed nests showed a correlation of 0.31. All other correlations had absolute values below 0.3.

Data from the UK and Germany were analysed separately. In each country we ran a hurdle regression model using the pscl package^90,91^ in R. Hurdle models are well suited to handle datasets with excess zeros, such as ours, since we have many records of host species with no parasitism. In Hurdle models, two different components are estimated: (i) a truncated count component and (ii) a hurdle component. The latter component estimates the zero vs. larger counts as a binomial process, while the former component excludes the zeros and models all the positive counts of parasitism. In our case a negative binomial distribution fitted the first component best. All predictor variables, 1–7 listed above, were part of both the binomial and the negative binomial components of the model and the model structure was the same in both cases.

In these hurdle models for UK and Germany, each passerine species breeding in the given country was included as a data point in our model. In each case, our response variable was the number of parasitized nests recorded. In these datasets we do not know the number of non-parasitized nests in the populations where these parasitism events were recorded. Therefore, we also included population size of each species in the given country (variable 8 listed above) to account for the uneven availability of the different species. All continuous predictor variables were log-transformed except overlap in breeding period. To create figures illustrating the significant effects, new models were run without non-significant predictor variables. To determine the model predictions from the hurdle models for UK and Germany, we used the predict function in R. We used the response predictions and kept all other variables constant whilst varying the focus variable within its observed range. For graphical purposes, these estimates were then scaled so that the highest estimate was the same as the highest estimate for predicted parasitism rates to allow comparison of patterns between the otherwise incomparable model estimates (rates vs numbers).

To determine the model predictions for the parasitism rates across Europe, we used the sim function in the arm package^92^ in R to simulate the posterior distributions of the parameters in our fitted model. From these distributions, average effect sizes and credibility intervals for different parameter combinations were calculated and used to make the figures. We chose the parasitism rates as reference values in our figures because these have an intuitive biological meaning while the predictions from the hurdle models provide a less obvious meaning biologically.

Next, we used the model predictions from the binomial model of parasitism rates as a species-specific index and a measure of suitability of each species as a cuckoo host. This index was scaled so that the most suitable species had an index of 1 and the least suitable species have an index of 0. Furthermore, we report the distribution of this host suitability index calculated for all European passerines (electronic supplementary material, Table S1). By separating between species where a corresponding cuckoo gens has been described based on egg characters^8–10,23,24^, we disclose how well our index relates to the number of parasitism cases found in the independent datasets for Germany and UK, and hence validates that these numbers do reflect parasitism rates.

## Electronic supplementary material

Table S1. Suitability index of all European passerine species
