# Supplementary material for: Characteristics determining host suitability for a generalist parasite
Source: Sci Rep. 2018 Apr 19;8:6285. doi: 10.1038/s41598-018-24627-1 (PMC5908913; doi:10.1038/s41598-018-24627-1)
Supplement: Supplementary file 1 — Table S1. Suitability index of all European passerine species [file 41598_2018_24627_MOESM1_ESM.doc]

**Characteristics determining host suitability for a generalist parasite**

Bård G. Stokke^*1,2^, Irja I. Ratikainen^1^, Arne Moksnes^1^, Eivin Røskaft^1^, Karl Schulze-Hagen^3^, David I. Leech^4^, Anders Pape Møller^5^ and Frode Fossøy^1,2^

*^1^Department of Biology, Faculty of Natural Sciences and Technology, Norwegian University of Science and Technology (NTNU), Høgskoleringen 5, NO-7491 Trondheim, Norway*

*^2^Norwegian Institute for Nature Research (NINA), P.O. Box 5685 Sluppen, NO-7485 Trondheim, Norway*

*^3^Bleichgrabenstr. 37, D-41063 Mönchengladbach, Germany*

*^4^British Trust for Ornithology, The Nunnery, Thetford, IP24 2PU, UK*

*^5^Ecologie Systématique Evolution, Université Paris-Sud, CNRS, AgroParisTech, Université Paris-Saclay, F-91405 Orsay Cedex, France*

**Author for correspondence* (bard.g.stokke@ntnu.no). ORCID ID: orcid.org/0000-0001-5589-6738. Phone: +47 73596128 / Fax: +47 73596100

**Table S1: Suitability index of all European passerine species.** The Suitability index quantifies how much the species suited as a host to the Common cuckoo, based on nest depth, nest type, nesting habitat, type of food it provides its young, size of the female and how much the nesting period overlaps with the cuckoo breeding period. Larger values indicate higher host suitability than lower values. See manuscript for details about methods for calculation. English and scientific species names are according to Birdlife International (<http://www.birdlife.org/>).

| Species |  | Suitability Index |
| --- | --- | --- |
| Olivaceous Warbler | Iduna pallida | 1,00 |
| Great Reed-warbler | Acrocephalus arundinaceus | 0,98 |
| Rufous-tailed Scrub-robin | Cercotrichas galactotes | 0,95 |
| Tawny Pipit | Anthus campestris | 0,94 |
| Orphean Warbler | Sylvia hortensis/crassirostris | 0,94 |
| Marsh Warbler | Acrocephalus palustris | 0,92 |
| Marmora's Warbler | Sylvia sarda | 0,92 |
| Common Reed-warbler | Acrocephalus scirpaceus | 0,91 |
| Little Bunting | Emberiza pusilla | 0,90 |
| Blyth's Reed-warbler | Acrocephalus dumetorum | 0,90 |
| Common Redstart | Phoenicurus phoenicurus | 0,90 |
| Zitting Cisticola | Cisticola juncidis | 0,89 |
| Great Grey Shrike | Lanius excubitor | 0,89 |
| White Wagtail | Motacilla alba | 0,89 |
| Water Pipit | Anthus spinoletta | 0,88 |
| Barred Warbler | Sylvia nisoria | 0,88 |
| European Robin | Erithacus rubecula | 0,85 |
| Meadow Pipit | Anthus pratensis | 0,85 |
| Subalpine Warbler | Sylvia cantillans | 0,85 |
| Sedge Warbler | Acrocephalus schoenobaenus | 0,84 |
| Lesser Whitethroat | Sylvia curruca | 0,84 |
| Dunnock | Prunella modularis | 0,84 |
| Western Yellow Wagtail | Motacilla flava | 0,83 |
| Wallcreeper | Tichodroma muraria | 0,83 |
| Black Redstart | Phoenicurus ochruros | 0,83 |
| Common Chiffchaff | Phylloscopus collybita | 0,83 |
| Melodious Warbler | Hippolais polyglotta | 0,83 |
| Corn Bunting | Emberiza calandra | 0,82 |
| Spectacled Warbler | Sylvia conspicillata | 0,82 |
| Cirl Bunting | Emberiza cirlus | 0,82 |
| Bluethroat | Cyanecula svecica | 0,82 |
| Sardinian Warbler | Sylvia melanocephala | 0,81 |
| Spanish Sparrow | Passer hispaniolensis | 0,81 |
| Woodlark | Lullula arborea | 0,80 |
| River Warbler | Locustella fluviatilis | 0,80 |
| Dartford Warbler | Sylvia undata | 0,80 |
| Red-backed Shrike | Lanius collurio | 0,80 |
| Tree Pipit | Anthus trivialis | 0,80 |
| Garden Warbler | Sylvia borin | 0,80 |
| Rock Pipit | Anthus petrosus | 0,80 |
| Western Bonelli's Warbler | Phylloscopus bonelli | 0,79 |
| Crested Lark | Galerida cristata | 0,79 |
| Wood Warbler | Phylloscopus sibilatrix | 0,78 |
| Red-throated Pipit | Anthus cervinus | 0,78 |
| Brambling | Fringilla montifringilla | 0,78 |
| Greater Short-toed Lark | Calandrella brachydactyla | 0,77 |
| Northern Wheatear | Oenanthe oenanthe | 0,77 |
| Yellow-breasted Bunting | Emberiza aureola | 0,77 |
| Whinchat | Saxicola rubetra | 0,76 |
| Common Nightingale | Luscinia megarhynchos | 0,76 |
| Greater Whitethroat | Sylvia communis | 0,76 |
| Citrine Wagtail | Motacilla citreola | 0,75 |
| Cetti's Warbler | Cettia cetti | 0,75 |
| Moustached Warbler | Acrocephalus melanopogon | 0,75 |
| Horned Lark | Eremophila alpestris | 0,75 |
| Rustic Bunting | Emberiza rustica | 0,74 |
| Snow Bunting | Plectrophenax nivalis | 0,74 |
| Ortolan Bunting | Emberiza hortulana | 0,74 |
| Eurasian Crag Martin | Ptyonoprogne rupestris | 0,73 |
| Common Stonechat | Saxicola torquatus | 0,73 |
| Short-toed Treecreeper | Certhia brachydactyla | 0,73 |
| Eurasian Blackcap | Sylvia atricapilla | 0,73 |
| European Serin | Serinus serinus | 0,73 |
| Common Grasshopper-warbler | Locustella naevia | 0,73 |
| Alpine Accentor | Prunella collaris | 0,72 |
| Lapland Longspur | Calcarius lapponicus | 0,72 |
| Woodchat Shrike | Lanius senator | 0,72 |
| Northern Wren | Troglodytes troglodytes | 0,72 |
| Olive-backed Pipit | Anthus hodgsoni | 0,72 |
| White-winged Snowfinch | Montifringilla nivalis | 0,71 |
| Eurasian Penduline-tit | Remiz pendulinus | 0,71 |
| Aquatic Warbler | Acrocephalus paludicola | 0,71 |
| Rock Bunting | Emberiza cia | 0,71 |
| Savi's Warbler | Locustella luscinioides | 0,71 |
| Greenish Warbler | Phylloscopus trochiloides | 0,70 |
| Calandra Lark | Melanocorypha calandra | 0,70 |
| Yellowhammer | Emberiza citrinella | 0,70 |
| Lesser Grey Shrike | Lanius minor | 0,69 |
| Icterine Warbler | Hippolais icterina | 0,68 |
| Blue Rock-thrush | Monticola solitarius | 0,68 |
| Rufous-tailed Rock-thrush | Monticola saxatilis | 0,68 |
| Spotted Flycatcher | Muscicapa striata | 0,68 |
| European Goldfinch | Carduelis carduelis | 0,68 |
| Twite | Linaria flavirostris | 0,68 |
| Common Firecrest | Regulus ignicapilla | 0,67 |
| Eurasian Golden Oriole | Oriolus oriolus | 0,67 |
| Grey Wagtail | Motacilla cinerea | 0,67 |
| Mistle Thrush | Turdus viscivorus | 0,67 |
| Reed Bunting | Emberiza schoeniclus | 0,67 |
| Citril Finch | Carduelis citrinella | 0,67 |
| Bearded Reedling | Panurus biarmicus | 0,67 |
| Thrush Nightingale | Luscinia luscinia | 0,66 |
| Goldcrest | Regulus regulus | 0,65 |
| Common Rosefinch | Carpodacus erythrinus | 0,65 |
| Common Linnet | Linaria cannabina | 0,64 |
| Ring Ouzel | Turdus torquatus | 0,64 |
| Willow Warbler | Phylloscopus trochilus | 0,64 |
| Common Chaffinch | Fringilla coelebs | 0,64 |
| Pine Grosbeak | Pinicola enucleator | 0,63 |
| European Greenfinch | Chloris chloris | 0,63 |
| Long-tailed Bushtit | Aegithalos caudatus | 0,63 |
| Eurasian Skylark | Alauda arvensis | 0,62 |
| Bohemian Waxwing | Bombycilla garrulus | 0,62 |
| Redwing | Turdus iliacus | 0,60 |
| Eurasian Treecreeper | Certhia familiaris | 0,60 |
| Iberian Azure-winged Magpie | Cyanopica cooki | 0,60 |
| Fieldfare | Turdus pilaris | 0,60 |
| Eurasian Bullfinch | Pyrrhula pyrrhula | 0,58 |
| Siberian Jay | Perisoreus infaustus | 0,57 |
| Eurasian Siskin | Spinus spinus | 0,56 |
| Redpoll | Acanthis flammea | 0,54 |
| Barn Swallow | Hirundo rustica | 0,54 |
| Two-barred Crossbill | Loxia leucoptera | 0,54 |
| Eurasian Blackbird | Turdus merula | 0,52 |
| White-throated Dipper | Cinclus cinclus | 0,52 |
| Scottish Crossbill | Loxia scotica | 0,51 |
| Red Crossbill | Loxia curvirostra | 0,50 |
| Parrot Crossbill | Loxia pytyopsittacus | 0,48 |
| Eurasian Magpie | Pica pica | 0,47 |
| Hawfinch | Coccothraustes coccothraustes | 0,47 |
| Song Thrush | Turdus philomelos | 0,47 |
| Eurasian Jay | Garrulus glandarius | 0,45 |
| Yellow-billed Chough | Pyrrhocorax graculus | 0,44 |
| Eurasian Tree Sparrow | Passer montanus | 0,40 |
| European Pied Flycatcher | Ficedula hypoleuca | 0,39 |
| Great Tit | Parus major | 0,38 |
| Rook | Corvus frugilegus | 0,36 |
| Red-billed Chough | Pyrrhocorax pyrrhocorax | 0,36 |
| House Sparrow | Passer domesticus | 0,35 |
| Northern House Martin | Delichon urbicum | 0,34 |
| Collared Sand Martin | Riparia riparia | 0,33 |
| Siberian Tit | Poecile cinctus | 0,33 |
| Marsh Tit | Poecile palustris | 0,33 |
| Collared Flycatcher | Ficedula albicollis | 0,33 |
| Crested Tit | Lophophanes cristatus | 0,32 |
| Willow Tit | Poecile montanus | 0,32 |
| Red-breasted Flycatcher | Ficedula parva | 0,32 |
| Rock Sparrow | Petronia petronia | 0,32 |
| Coal Tit | Periparus ater | 0,32 |
| Eurasian Blue Tit | Cyanistes caeruleus | 0,32 |
| Eurasian Nuthatch | Sitta europaea | 0,31 |
| Northern Nutcracker | Nucifraga caryocatactes | 0,30 |
| Carrion Crow | Corvus corone | 0,28 |
| Common Starling | Sturnus vulgaris | 0,25 |
| Eurasian Jackdaw | Corvus monedula | 0,05 |
| Common Raven | Corvus corax | 0,00 |
